# Surgery on primary tumor shows survival benefit in selected stage IV colon cancer patients: A real-world study based on SEER database

**DOI:** 10.7150/jca.43518

**Published:** 2020-03-15

**Authors:** Jing Xu, Tai Ma, Yuanzi Ye, Zhipeng Pan, Donghui Lu, Faming Pan, Wanren Peng, Guoping Sun

**Affiliations:** 1 Department of Oncology, The First Affiliated Hospital of Anhui Medical University, Hefei, Anhui Province 230000, China; 2 Department of Pathology, The First Affiliated Hospital of Anhui Medical University, Hefei, Anhui Province 230000, China; 3 Department of Radiology, The 901st Hospital of the Joint Logistics Support Force of PLA, Hefei, Anhui Province 230031, China; 4 Department of Epidemiology and Biostatistics, School of Public Health, Anhui Medical University, Hefei, Anhui Province 230000, China

## Abstract

**Objectives**: Most patients with stage IV colon cancer did not have the opportunity for curative surgery, only selected patients could benefit from surgery. This study aimed to determine whether surgery on the primary tumor (SPT) should be performed in patients with stage IV colon cancer and how to select patients for SPT.

**Methods**: This study included 48,933 patients with stage IV colon cancer who were identified in the Surveillance, Epidemiology and End Results (SEER) database between 1998 and 2015. Propensity score matching (PSM) analysis was adopted to balance baseline differences between SPT and non-surgery groups. Kaplan-Meier (K-M) curves were utilized to compare the overall survival (OS). Prognostic nomograms were generated to predict survival based on pre- and post-operative risk factors. Patients were divided into low, middle, and high mortality risk subsets for OS by X-tile analyses based on scores derived from above nomograms.

**Results**: Patients with SPT had a significantly longer OS than those without surgery, regardless of the metastatic sites and diagnostic years. Nomograms, according to the pre- and post-operative risk factors, showed moderate discrimination (all C-indexes above 0.7). Based on X-tile analyses, low mortality risk subset (post-operative score ≤ 22.3, preoperative score ≤ 9.7) recommended for SPT, and high mortality risk was not.

**Conclusions**: SPT led to prolonged survival in stage IV colon cancer. Our nomograms would help to select suitable patients for SPT.

## Background

Colon cancer is the second leading cause of cancer-related death worldwide^1^. The incidence and mortality rate of colon cancer has decreased in the past few decades due to the earlier diagnosis and improvement of therapy. However, most patients with stage IV cancer did not have the opportunity for curative surgery, only selected patients could benefit from surgery^1^. In the National Comprehensive Cancer Network (NCCN) guidelines, it was not recommended to remove the primary tumor if the primary lesion was not in the risk of obstruction, bleeding, or perforation^2^,^3^.

The effectiveness of surgery on the primary tumor (SPT) in stage IV colon cancer was controversial^4^,^5^,^6^,^7^,^8^,^9^. The first-line therapy for stage IV colon was fluorouracil-based chemotherapy combined with targeted therapy^10^, surgical intervention was only considered in case of tumor-related complications such as bleeding, perforation, and obstruction. Surgery was also performed in the setting in which all visible tumor can be removed^11^. Scoggins et al. observed no significant survival benefit associated with surgery for stage IV colorectal cancer^12^ .Tebbutt et al. found that the rate of major intestinal complications such as obstructions was lower in patients receiving chemotherapy than in those who underwent primary resection^13^. Hu et al. reported that the resection rate of stage IV colorectal cancer decreased from 74.5% in 1988 to 57.4% in 2010^7^. The relative survival rate rose from 8.6% to 17.8%, and this tendency was more pronounced in 2001-2010. They suggested that this trend was due to the emergence of multiple treatment options for colorectal cancer after 2001, as well as the decreased primary tumor resection rate. Thus, it was concluded that the prolongation of survival in stage IV colon cancer patients was not associated with SPT.

On the other hand, an increasing number of studies demonstrated the value of SPT in stage IV colon cancer. Cook et al.^14^ retrospectively analyzed the statistics of advanced colorectal cancer in the United States from 1998 to 2000, in which the initial resection rate was as high as 66%. The 1-year survival rate of patients with SPT was much higher than that of those without SPT. Ruo et al.^15^ found that for asymptomatic and incurable advanced colorectal cancer, the surgical removal of primary lesions significantly prolonged the median survival. A population-based study of the British Columbia Cancer Agency indicated that palliative surgery on the primary site of stage IV colorectal cancer was associated with longer survival^16^. Meta-analyses also showed that SPT could prolong the survival in stage IV colorectal cancer^17^-^20^. Nevertheless, most of those studies were retrospective, single-center designed. The number of patients in meta-analyses performed based on the previous studies was relatively small (less than 16,295) compared to that in real-world practice. However, due to the high heterogeneity of the study populations and severe selection bias, their results were not entirely convincing.

There were many reasons why these studies raised different opinions of SPT in stage IV colon cancer, including study design, study population, and the quality of clinical data. Besides, for studies which recommended SPT in stage IV colon cancer, they did not give recommendations to select appropriate patients for SPT. To the best of our knowledge, no study has identified how to select patients for surgery on the primary site of stage IV colorectal cancer.

The Surveillance, Epidemiology and End Results (SEER) database is a real-world database of the United States which covered approximately 28% of the American population. In the SEER database, the clinical characteristics, as well as treatments and survival information of colorectal cancer were recorded. We used data of stage IV colon cancer in the SEER database from 1974 to 2015 to explore the value of SPT on stage IV colon cancer patients and develop nomograms to help select patients who were suitable for SPT.

## Materials and Methods

### Database and patient selection

The SEER 18 registries research database (1974-2015) was used to identify patients with stage IV colon cancer. The selection criteria included: 1.Year of diagnosis: 1974 to 2015, 2. Primary colon cancer (International Classification of Diseases [ICD]-0-3/ World Health Organization 2008), excluding rectal cancer and appendix cancer. 3. Malignancy (behavior code ICD-0-3), and 4.Stage M1 based on the American Joint Committee on Cancer/Union for International Cancer Control (AJCC/UICC) staging system. The following variables were collected: 1. Baseline characteristics of patients: diagnosis age, gender, and race, 2. Pathology of tumor: histology/behavior code for analysis(ICD-0-3), anatomical site of primary tumor, differentiation grade, T, N, and M stages according to the 3^rd^, 6^th^ or 7^th^ edition AJCC/UICC staging system,3. Tumor size, regional lymph node (N) status before surgery, tumor biomarker CEA status, numbers of N examined and numbers of N positive after surgery, and different distant metastatic sites, e.g., liver, lung, bone, and brain, 4. Therapy: surgery code for the primary site, surgery on distant sites and chemotherapy (yes or no), and 5. Survival information: survival month and SEER death classification. Patients with unknown surgery status, combined primary and distant site surgery, local tumor destruction (e.g., photodynamic therapy, electrocautery, fulguration, cryosurgery, and laser), local tumor excision, or unknown information on the above variables were excluded.

### Statistical analysis

A Chi-squared test was utilized to evaluate the differences of baseline characteristics between the SPT and non-surgery groups. To balance these characteristics, patients of these two groups were matched using the propensity score matching (PSM) analysis^21^. One-to-one matching was carried out without replacement, and a caliper width of 0.0003 was adopted for the overall population. The Kaplan-Meier (K-M) method and a log-rank test were performed to compare the overall survival (OS) between the score-matched groups.

The nomograms based on pre- and post-operative risk factors were generated for survival prediction. The accuracy of the nomogram model was assessed using discrimination and calibration. The nomograms were subjected to 1,000 bootstraps resamples for the calculation of the estimated Harrell's concordance index (C-index) as an index of model performance^22^. Moreover, graphic calibration curves were created to represent the discrepancy between the observed outcome frequencies and predicted probabilities. Based on the scores from nomograms, patients were divided into different mortality risk subsets by X-tile analysis^23^.

Most statistical analyses were performed using SPSS statistical software package version 22.0 (IBM Corporation, Armonk, NY, USA). The nomograms and validation procedures were performed using Rstudio software based on 'R' version 3.5.0. The X-tile analysis was conducted by X-tile software. Differences were considered statistically significant at P <0.05. All P values were 2-tailed.

## Results

### Overall analysis comparing OS between the SPT and non-surgery groups

**Figure 1:**
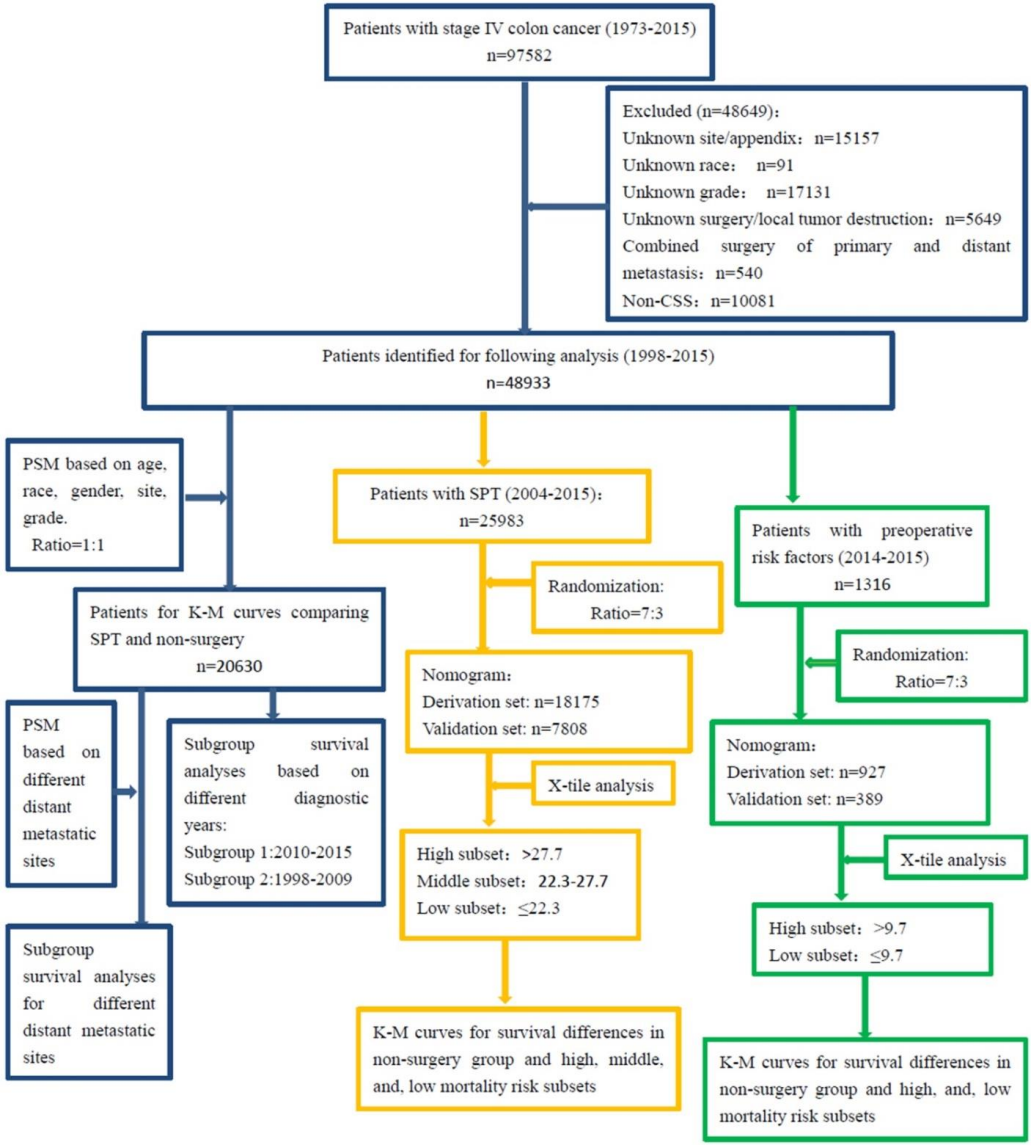
** Flow diagram of patient selection and study development.** PSM: propensity score-match analysis, K-M: Kaplan-Meier; OS: overall survival.

**Figure 1** showed the patient selection process. A total of 97,582 patients diagnosed with stage IV colon cancer were identified from 1974 to 2015, and 48,649 were excluded as they met the exclusion criteria. A total of 25,983 patients underwent SPT while the others did not undergo surgery. And their diagnostic years were from 1998 to 2015.

**Figure 2:**
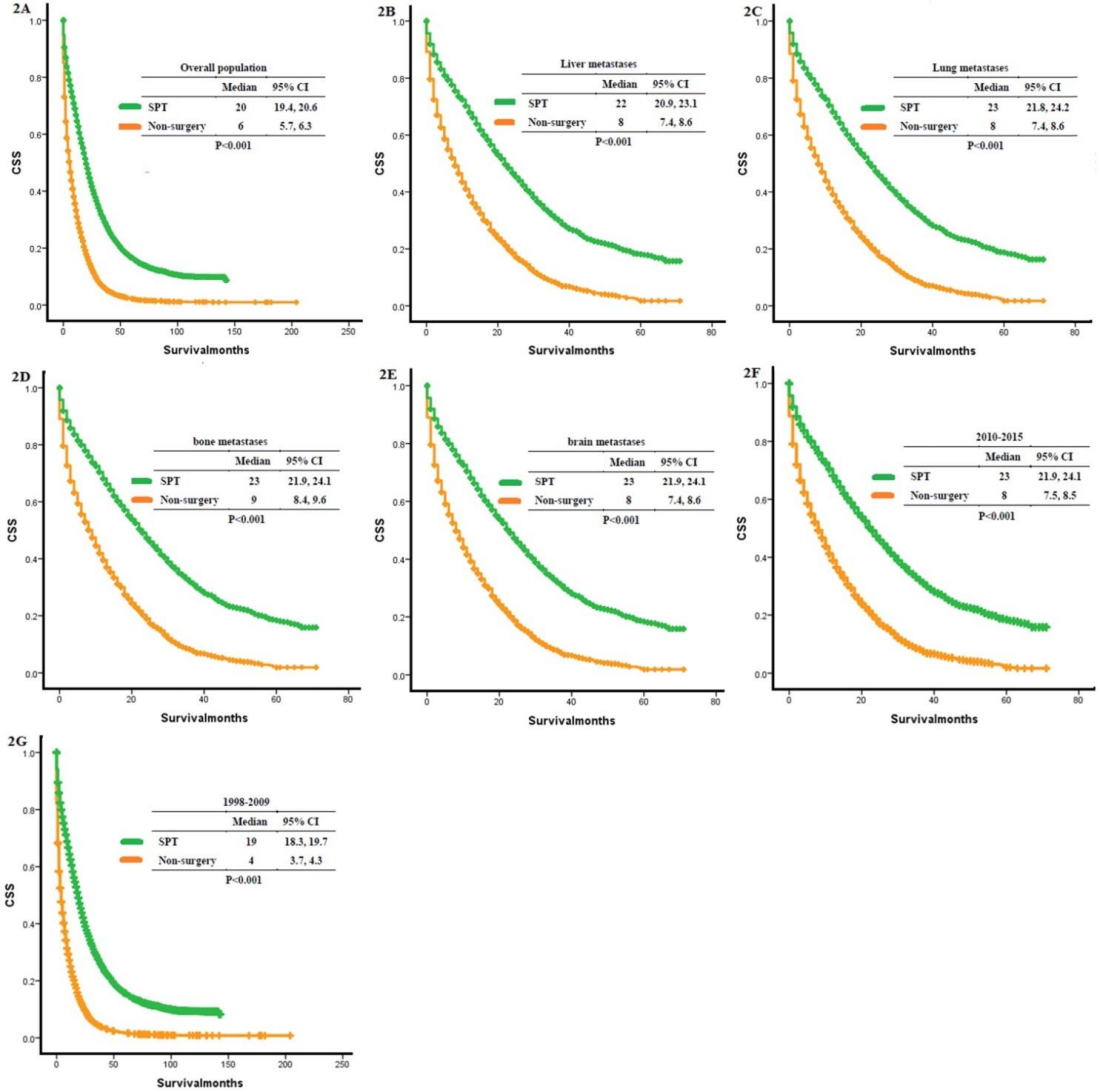
** Kaplan-Meier curves for overall survival.** (A) Overall analysis of OS: SPT vs. On-surgery, (B) distant metastasis to liver: SPT vs. non-surgery, (C) distant metastasis to lung: SPT vs. non-surgery, (D) distant metastasis to bone: SPT vs. non-surgery, (E) distant metastasis to brain: SPT vs. non-surgery, (F) patients diagnosed between 2010 and 2015: SPT vs. non-surgery, (G) patients diagnosed between 1998 and 2009: SPT vs. non-surgery. SPT, surgery on primary tumor; OS: overall survival.

The mean age of the whole population was 65.26 ± 13.868 years (mean ± SD). Therefore, patients were divided into the following two groups according to age: ≤ 65 and > 65 years. Chi-squared tests showed significant differences in baseline characteristics of patients, such as age, gender, race, site, and grade **(Table 1)**. 20,630 patients were matched between the SPT group and the non-surgery group by PSM analysis. Patients with SPT had a significantly longer OS than those without surgery (median OS: 20 months vs. 6 months, P < 0.001, **Figure 2A**).

### Subgroup analyses comparing OS between the SPT and non-surgery groups of different metastatic sites and diagnostic years

We matched the patients of stage IV colon cancer patients according to different metastatic sites **(Supplemental Table 1)**. The SPT group remained to show longer OS compared to the non-surgery group (liver: 7592 patients, median OS: 22 months vs. 8 months, P <0.001, **Figure 2B**; lung: 7786 patients, median OS: 23 months vs. 8 months, p<0.001, **Figure 2C**; bone: 8400 patients, median OS: 23 months vs. 9 months, p<0.001, **Figure 2D**; brain: 8400 patients, median OS: 23 months vs. 8 months, p<0.001, **Figure 2E**).

As surgical technique and technology have improved over the time frame of the study period (1998-2015), we re-analyzed the data according to district time periods (2010-2015, 1998-2009) to allow for percolation of advances in surgery and surgical technology to involve the broadest swath of these providers. We found that patients with SPT had prolonged OS than the non-surgery group in different time periods (2010-2015: 8791 patients, median OS: 25 months vs. 8 months, P <0.001, **Figure 2F**; 1998-2009: 11839 patients, median OS: 19 months vs. 4 months, p<0.001, **Figure 2G**).

### Nomogram constructions for predicting OS in SPT group

A total of 25983 patients underwent SPT. After randomization, 18175 patients were selected for the derivation set, while 7808 patients were included in the validation set. **Table 2** showed the results of multivariate analyses of overall patients and the derivation set. The following tumor features were connected independently with OS: age, site, grade, T stage, N examined, N positive, and chemotherapy.

**Figure 3:**
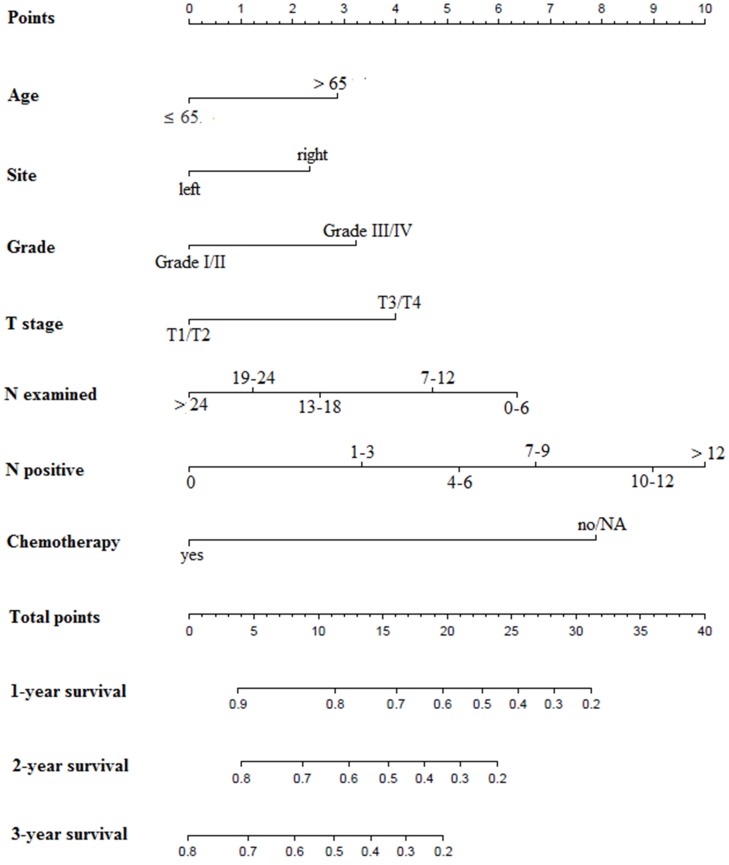
** Prognostic nomograms for patients with surgery on primary tumor.** N: regional lymph node; NA: not available.

**Figure 4:**
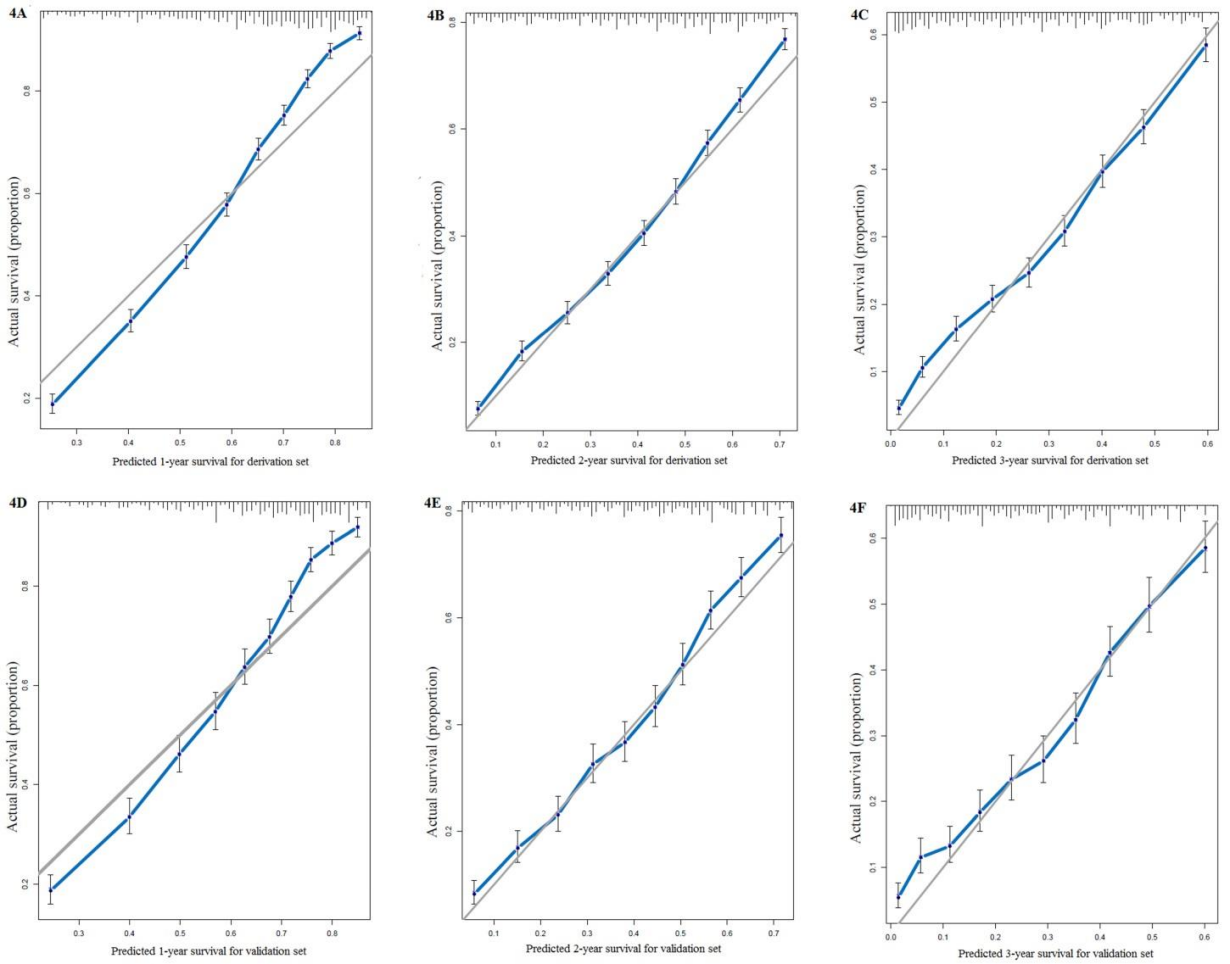
Calibration curves for survival prediction of patients with surgery on primary tumor at (A) 1year, (B) 2years and (C) 3years in the derivation set, and calibration curves for the prediction of patient survival at (D) 1year, (E) 2years and (F) 3years in the validation set.

We used all the significant independent factors to create a prognostic nomogram for the derivation group (**Figure 3**). The C-index in this group was 0.715, with optimal discrimination. The calibration plots for the probability of survival indicated moderate agreement between the nomogram prediction and actual observation (**Figures 4A-C**).

The C-index of the validation set for the prediction of OS was 0.714. And calibration curves showed good agreement of OS between prediction and observation (**Figures 4D-F**).

Due to the improvement of systemic therapy and surgical technology, we also calculated the accuracy of the predictive nomogram model in patients diagnosed from 2010-2015. The C-index of these 11886 patients was 0.74.

### X-tile analysis for dividing SPT group into different mortality risk subset

**Figure 5:**
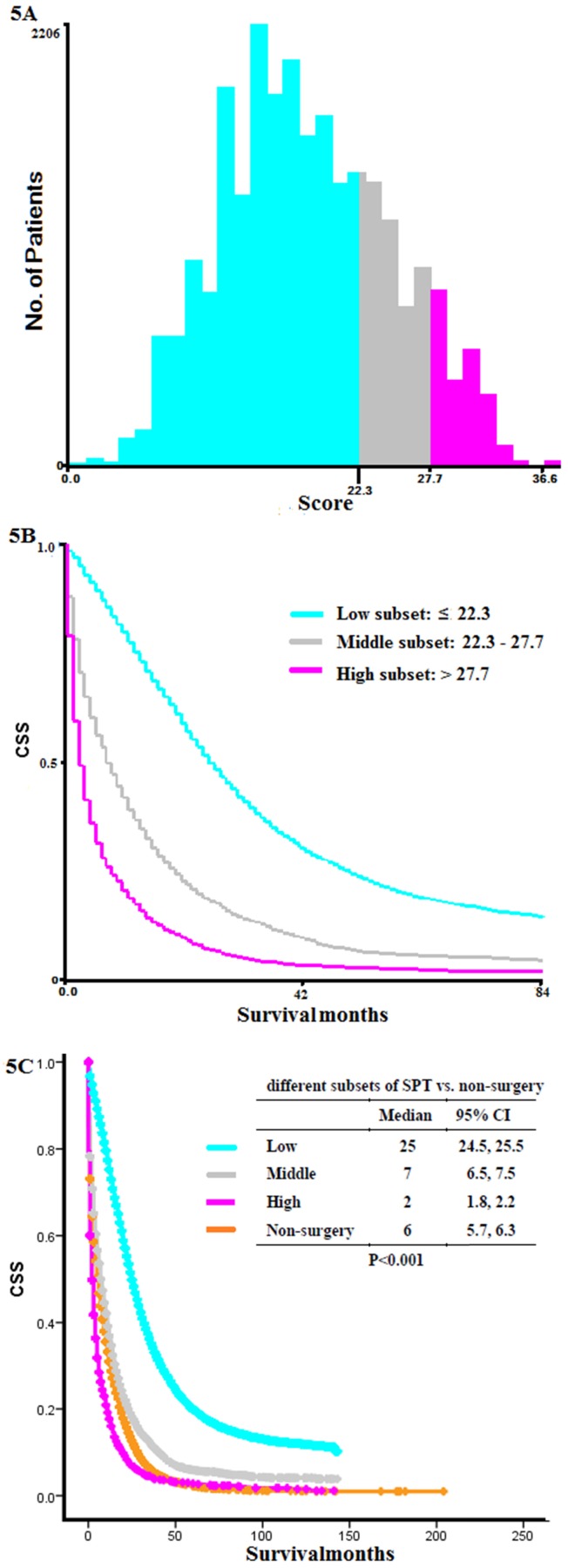
** X-tile analysis and Kaplan-Meier curves for overall survival.** (A) Numbers of patients with SPT in high, middle, and low mortality risk subsets, (B) K-M curves for OS in high, middle, and low mortality risk subsets, (C) K-M curves for OS in different subsets of SPT group and non-surgery group. SPT, surgery on primary tumor; K-M: Kaplan-Meier; OS: overall survival.

We calculated the total scores of each patient from the SPT group based on the previous nomogram. According to X-tile analysis, patients scored below 22.3 were considered in low mortality risk subset, those scored above 27.7 were considered in high mortality risk subset, and those scored between 22.3 and 27.7 were in the middle (**Figure 5A-B**). K-M curve showed that the OS of patients in high mortality risk subset was even shorter than those without surgery (low vs. middle vs. high vs. non-surgery: 25 months vs. 7 months vs. 2 months vs. 6 months, P < 0.001, **Figure 5C**). Patients categorized in low mortality risk subset benefitted most from SPT.

### Nomogram constructions based on preoperative risk factors

**Figure 6:**
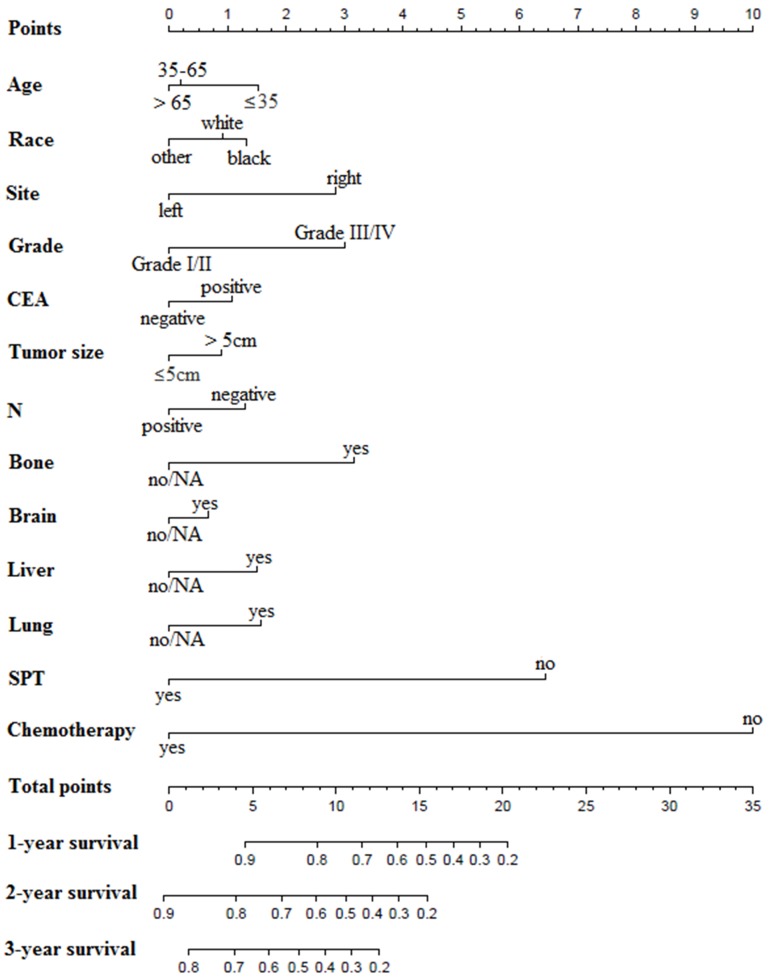
** Prognostic nomograms based on preoperative risk factors.** CEA: carcinoembryonic antigen; N: regional lymph node; SPT, surgery on primary tumor; NA: not available.

**Figure 7:**
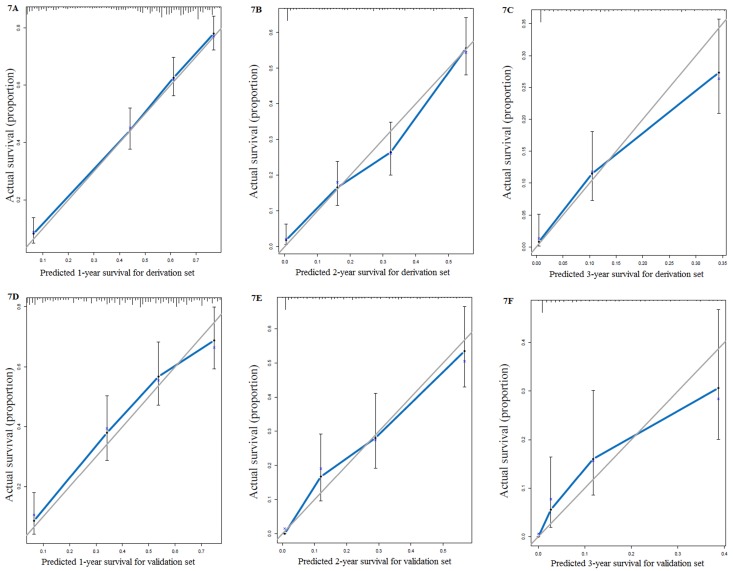
Calibration curves for nomograms based on preoperative risk factors: (A) 1 year, (B) 2 years and (C) 3 years in the derivation set. AND calibration curves for the prediction of patient survival at (D) 1 year, (E) 2 years and (F) 3 years in the validation set.

All the preoperative risk factors were listed in **Table 3**, such as age, gender, race, site, grade, CEA, tumor size, regional lymph node metastases, different distant metastases, and treatment choices. 1316 patients diagnosed between 2014 and 2015 with stage IV colon cancer provided detailed data of these factors. Multivariate analyses revealed that age, race, site, grade, CEA, tumor size, N, different distant metastases, and treatment choices were independently related to OS. By randomization of the 7:3 ratio, 927 patients were chosen for the derivation set and the rest for the validation set. The nomogram for the derivation set was exhibited in **Figure 6**. The C-index showed moderate discrimination (derivation set: 0.767; validation set: 0.742). And calibration curves suggested that the nomogram prediction had good agreement with actual observation (**Figures 7A-F**).

**Figure 8:**
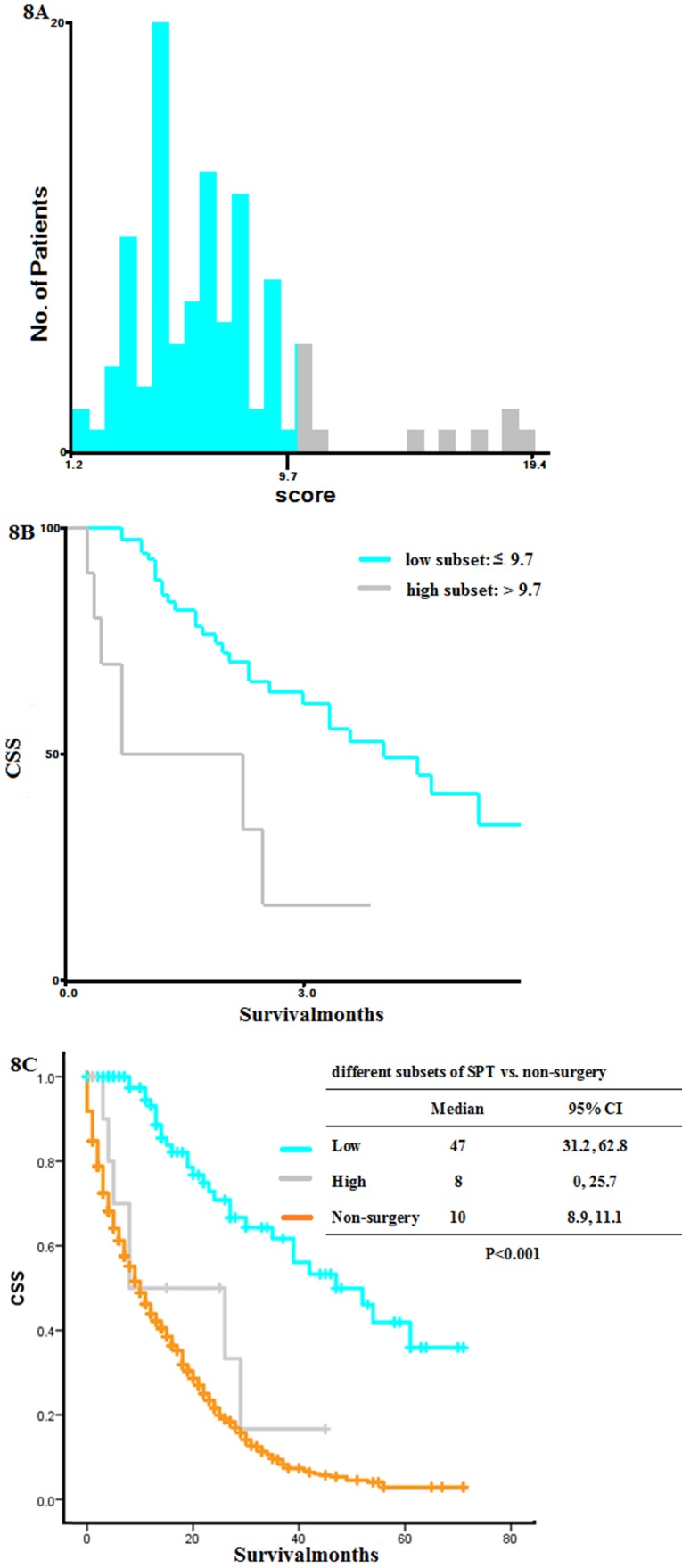
** X-tile analysis based on the preoperative nomogram and Kaplan-Meier curves for overall survival.** (A) Numbers of patients at diagnosis in high and low mortality risk subsets, (B) K-M curves for OS in high and low mortality risk subsets, (C) K-M curves for OS in high and low risk subsets compared with that in non-surgery group. SPT, surgery on primary tumor; K-M: Kaplan-Meier; OS: overall survival.

We divided these patients into high and low mortality risk subsets using X-tile analysis. Patients with a score below 9.7 were put into low mortality risk subset, while those 9.7 were in high mortality risk subset (**Figure 8A-B**). K-M curves indicated that patients in high mortality risk subset had shorter OS than those without surgery (low vs. high vs. non-surgery: 47 months vs. 8 months vs. 10 months, P < 0.001, **Figure 8C**). So SPT was recommended to patients below 9.7 at diagnosis.

## Discussion

There was an ongoing debate about surgery on primary tumors in patients with primary stage IV colon cancer. In the current population-based study, we identified those patients with SPT had a significantly longer OS than those without surgery, regardless of the metastatic sites and diagnostic years (**Figure 2**). According to X-tile analysis, which divided the SPT group into different mortality risk subset, patients with SPT scored below 22.3 were considered in low mortality risk, and those above 27.7 were considered in high mortality risk. At diagnosis, patients with a preoperative score below 9.7 were considered in low mortality risk, and those above 9.7 were considered in high mortality risk. Patients in high mortality risk subset might have more reduced survival than those without surgery. In contrast, patients with low mortality risk had prolonged survival.

Treatment for patients with initial stage IV colon cancer required careful discussion of expected overall survival outcomes, complications from treatment modalities, and quality of life after treatment. The NCCN guidelines recommend operative intervention on metastatic patients in certain cases. For example, they left room for converting unresectable to resectable disease after chemotherapy, or for staged resection in the face of resectable metastases. Nevertheless, for patients with truly unresectable metastases, they did not recommend palliative SPT based on the following considerations: first, some unresectable metastatic disease presented with asymptomatic primary tumor. Second, other symptomatic tumors could achieve symptomatic improvement by chemotherapy in combination with mono antibodies within the first 1 to 2 weeks^24^. Third, there was no high-grade evidence supporting palliative surgery. However, these recommendations were proposed based on lower-level evidence. For example, a systemic review of 798 studies published until January 2012 found that SPT did not reduce the risk of complications due to the primary tumor. They also raised concerns about the quality of published literature and called for high-quality data to help address this issue. The severe chemotherapy-related morbidity and mortality, as well as the study data on clinical efficacy, also made non-surgery management for stage IV colorectal cancer very controversial^10^.

The current study included data from 48,933 patients in a real-world population-based SEER database. We used KM analysis with PSM to show the improved survival after SPT in patients with primary stage IV colon cancer. Several retrospective analyses also indicated the survival benefits of SPT in patients with stage IV colorectal cancer 25-28. Their sample sizes were relatively small (ranges from 208 patients to 1,982 patients). To the best of our knowledge, only one study identified 37,793 metastatic colorectal cancer patients in the SEER database from 1998 to 2009. However, they did not conduct detailed analyses to identify patients who should be recommended for surgery. Molecular targeted therapeutic drugs such as bevacizumab and cetuximab were applied in the treatment of stage IV colorectal cancer since 2010, which had a significant impact on the survival outcome^29^. In addition, data on a larger number of patients with detailed AJCC 7^th^ edition staging information and metastases have been added to the SEER database since 2010. Our study included more up-to-date data from a large cohort of stage IV colorectal cancer patients in the SEER database between 1974 and 2015, and detailed analyses of the different metastatic sites were feasible in the current study.

Although our results showed a better survival outcome for SPT in stage IV colorectal cancer, not all patients would benefit from SPT. Based on the results of X-tile analyzes, we suggested that patients in the SPT group should be explicitly divided into high, middle, and low mortality risk subsets based on postoperative factors and high and low-risk subsets on preoperative ones. Patients in high mortality risk subset (scored more than 27.7 by postoperative nomogram, and more than 9.7 by preoperative nomogram) had a much shorter OS than those without surgery. This finding reminded surgeons that patients with SPT scored below 22.3 by postoperative nomogram or below 9.7 by preoperative nomogram were suitable patients for SPT. Nevertheless, for those sored more than 27.7 by postoperative nomogram or 9.7 by preoperative nomogram, SPT was not recommended. With the preoperative nomogram, we answered the question of how to predict the OS of patients at diagnosis. The treatment choices by oncologists had a significant prognosis in survival. However, the sample size in the preoperative analysis was relatively small, and further study of large sample size and high-quality data will help to validate the preoperative nomogram.

For some stage IV colon cancer patients without SPT at diagnosis, they might require emergency surgery to manage these complications^5^, ^30^-^32^. However, postoperative mortality rate of emergency surgery was much higher than that of elective surgery^5^,^33^-^35^. Patients who underwent emergency surgery were supposed to have more surgical complications than those with SPT^35^. It was important to select patients with stage IV colon cancer to have better surgery management considering the relevant complications associated with the surgery itself, the recovery of digestive system function and ostomy care. We found that in patients who underwent SPT, certain features such as younger age, left colon cancer site, well- and moderate-differentiated tumor, T1/T2 stage, more N examined, less N positive, and chemotherapy were significantly related to better prognoses. A growing body of evidence suggested that primary tumor locations would be a valuable prognostic factor^36^ , and colorectal cancers of different location have specific epidemiological and clinicopathological characteristics^37^. Our results showed that patients with left-sided tumors had a significantly increased survival, which was consistent with findings from previous studies. Also, patients with left-sided colorectal tumors who underwent SPT had better clinical outcomes than other subgroups.

The major strengths of our current study include a large sample size of stage IV colorectal cancer patients identified in the population-based SEER database from 1998 to 2015. Detailed data collected since 2010, the analyses of SPT score, and predictive nomograms using preoperative characteristics added more evidence to SPT as a recommendation for selected stage IV colorectal cancer patients. Nevertheless, our study had some limitations. The SEER registry did not provide data on performance status, tumor-related complications, symptoms, basic diseases, chemotherapy regimens, and distinction of emergency and elective surgery. Therefore, these potential prognostic factors were not included in our analyses. In addition, our results might result from potential selection bias. The patients offered surgery might be those with limited comorbidities, or those with resectable disease, or those responded well to chemotherapy. However, the C-index of the predictive nomogram models in the current study were all higher than 0.7, which indicated that modeling analyses using characteristics in the current SEER database would be clinically used in the decision-making process. Furthermore, surgery might be possible in resectable patients.

In conclusion, SPT could improve the survival of patients with stage IV colon cancer, regardless of the metastatic types. And our nomograms could help to select suitable patients with stage IV colorectal cancer for SPT in the decision-making process.

## Supplementary Material

Supplementary table S1.

## Figures and Tables

**Table 1:** Baseline characteristics of all patients with stage IV colon cancer and propensity score-matched patients.

Characteristic	All patients				PSM patients		
	SPT (n=38618) No. of patient (%)	Non-surgery (n=10315) No. of patient (%)	P		SPT (n=10315) No. of patient (%)	Non-surgery (n=10315) No. of patient (%)	P
**Age**			<0.001				1.000
≤65	19453 (50.4%)	4844 (47.0%)			4844 (47.0%)	4844 (47.0%)	
>65	19165 (49.6%)	5471 (53.0%)			5471 (53.0%)	5471 (53.0%)	
**Gender**			<0.001				1.000
Male	19382 (50.2%)	5516 (53.5%)			5516 (53.5%)	5516 (53.5%)	
Female	19236 (49.8%)	4799 (46.5%)			4799 (46.5%)	4799 (46.5%)	
**Race**			<0.001				1.000
White	29851 (77.3%)	7710(74.7%)			7710 (74.7%)	7710 (74.7%)	
Black	5680 (14.7%)	1753 (17.0%)			1753 (17.0%)	1753 (17.0%)	
Other	3087 (8.0%)	852 (8.3%)			852 (8.3%)	852 (8.3%)	
**Site**			<0.001				1.000
Left	14400 (37.3%)	4283 (41.5%)			4283 (41.5%)	4283 (41.5%)	
Right	24218 (62.7%)	6032 (58.5%)			6032 (58.5%)	6032 (58.5%)	
**Grade**			<0.001				1.000
Grade I/II	25408 (65.8%)	7212 (69.9%)			7212 (69.9%)	7212 (69.9%)	
Grade III/IV	13210 (34.2%)	3103 (30.1%)			3103 (30.1%)	3103 (30.1%)	

PSM: propensity score-matched; Left: descending colon and sigmoid colon; Right: cecum, ascending colon, hepatic flexure, transverse colon and splenic flexure.

**Table 2:** Multivariate analyses of cancer-specific survival in patients with SPT.

Characteristic	All patients				Derivation set		
	No. of patient (n=25983)	HR (95% CI)	P		No. of patient (n=18175)	HR (95% CI)	P
**Age**			<0.001				<0.001
≤65	13740	1			9545	1	
>65	12243	1.061 (1.029, 1.093)			8630	1.341 (1.293, 1.389)	
**Gender**			0.738				0.189
Male	13155	1			9177	1	
Female	12898	0.995 (0.967, 1.024)			8998	0.977 (0.944, 1.011)	
**Race**			0.083				<0.001
White	19868	1			13948	1	
Black	3945	1.034 (0.993, 1.077)			2689	1.095 (1.043, 1.149)	
Other	2170	0.965 (0.915, 1.018)			1538	0.932 (0.875, 0.993 )	
**Site**			0.004				<0.001
Left	9552	1			6679	1	
Right	16431	1.047 (1.015,1.080)			11496	1.245 (1.199, 1.291)	
**Grade**			<0.001				<0.001
Grade I/II	17146	1			11945	1	
Grade III/IV	8837	1.070 (1.038, 1.104)			6230	1.378 (1.328, 1.429)	
**T stage**			0.001				<0001
T1/T2	896	1			634	1	
T3/T4	25087	1.150 (1.055, 1.254)			17541	1.475 (1.332, 1.633)	
**N examined**			<0.001				<0.001
0-6	2658	1			1844	1	
7-12	6015	0.927 (0.880, 0.977)			4192	0.848 (0.797, 0.903)	
13-18	8156	0.831 (0.790, 0.874)			5748	0.685 (0.645, 0.728)	
19-24	4425	0.776 (0.733, 0.821)			3094	0.602(0.563, 0.644)	
>24	4729	0.747(0.706, 0.790)			3297	0.538 (0.503,0.575)	
**N positive**			<0.001				<0.001
1-3	7782	1			5421	1	
4-6	5264	1.083 (1.039, 1.128)			3734	1.205 (1.147, 1.266)	
7-9	3154	1.132 (1.078, 1.188)			2233	1.400 (1.321, 1.483)	
10-12	1926	1.192 (1.126, 1.263)			1334	1.754 (1.637, 1.879)	
>12	3420	1.251 (1.192, 1.313)			2375	1.914 (1.806, 2.028)	
0	4437	0.871(0.831, 0.913)			3078	0.723 (0.684, 0.765)	
**Chemotherapy**							<0.001
Yes	16294	1	<0.001		11394	1	
No/NA	9689	1.116 (1.083, 1.151)			6781	2.140 (2.064, 2.220)	

Left: descending colon and sigmoid colon; Right: cecum, ascending colon, hepatic flexure, transverse colon and splenic flexure; N: lymph node; NA: not available.

**Table 3:** Multivariate analyses of overall survival based on preoperative risk factors.

Characteristic	All patients				Derivation set		
	No. of patient (n=1316)	HR (95% CI)	P		No. of patient (n=927)	HR (95% CI)	P
**Age**			0.103				0.679
≤35	34	1			23	1	
35-65	738	0.728 (0.481, 1.102)			534	0.807 (0.486, 1.340)	
>65	544	0.825 (0.540, 1.262)			370	0.791 (0.469, 1.333)	
**Gender**			0.454				0.129
Male	751	1			512	1	
Female	565	0.949 (0.827, 1.089)			415	0.879 (0.743, 1.038)	
**Race**			0.004				0.370
White	965	1			690	1	
Black	231	1.070 (0.899, 1.272)			152	1.091 (0.881, 1.352)	
Other	120	0.672 (0.522, 0.864)			85	0.853 (0.635, 1.147)	
**Site**			<0.001				<0.001
Left	600	1			421	1	
Right	716	1.471 (1.275, 1.697)			506	1.599 (1.338, 1.911)	
**Grade**			<0.001				<0.001
Grade I/II	979	1			700	1	
Grade III/IV	337	1.649 (1.413, 1.926)			227	1.649 (1.367, 1.990)	
**CEA**			0.001				0.117
Positive	1137	1			802	1	
Negative	179	0.708 (0.575, 0.871)			125	0.822 (0.642, 1.051)	
**Tumor size**			0.005				0.110
≤5cm	732	1			521	1	
>5cm	584	1.219 (1.062, 1.399)			406	1.145 (0.970, 1.351)	
**N**			0.025				0.021
Positive	579	1			390	1	
Negative	737	1.175 (1.021, 1.352)			537	1.223 (1.031, 1.451)	
**Liver**			0.057				0.045
Yes	1080	1			764	1	
No/NA	236	0.836 (0.695, 1.005)			163	0.796 (0.636, 0.995)	
**Lung**			<0.001				0.004
Yes	373	1			256	1	
No/NA	943	0.762 (0.657, 0.885)			671	0.768 (0.642, 0.919)	
**Bone**			<0.001				0.001
Yes	101	1			73	1	
No/NA	1215	0.646 (0.507, 0.822)			854	0.613 (0.457, 0.822)	
**Brain**			0.060				0.660
Yes	25	1			16	1	
No/NA	1291	0.659 (0.426, 1.018)			911	0.881 (0.499, 1.553)	
**SPT**			<0.001				<0.001
Yes	106	1			79	1	
No	1210	3.135 (2.223, 4.422)			848	3.026 (2.024, 4.523)	
**Chemotherapy**			<0.001				<0.001
Yes	979	1			690	1	
No/NA	337	4.343 (3.688, 5.114)			237	5.246 (4.302, 6.396)	

Left: descending colon and sigmoid colon; Right: cecum, ascending colon, hepatic flexure, transverse colon and splenic flexure; N: lymph node; NA: not available.
