# Supplementary material for: Surgery on primary tumor shows survival benefit in selected stage IV colon cancer patients: A real-world study based on SEER database
Source: J Cancer. 2020 Mar 15;11(12):3567–79. doi: 10.7150/jca.43518 (PMC7150453; doi:10.7150/jca.43518)
Supplement: Supplementary file 1 — Supplementary table S1. [file jcav11p3567s1.pdf]

Supplemental Table 1. propensity score-matching analyses based on different metastatic sites

Metastatic site	Before PSM		P	After PSM		P
	SPT No. of patient (%)	Non-surgery No. of patient (%)		SPT No. of patient (%)	Non-surgery No. of patient (%)	
**Liver**			<0.001			1.000
Yes	2927 (69.7%)	3722 (81.1%)		2927 (77.1%)	2927 (77.1%)	
No/NA	1273 (30.3%)	869 (18.9%)		869 (22.9%)	869 (22.9%)	
**Lung**			<0.001			1.000
Yes	629 (15.0%)	1327 (28.9%)		629 (16.2%)	629 (16.2%)	
No/NA	3571 (85.0%)	3264 (71.1%)		3264 (83.8%)	3264 (83.8%)	
**Bone**			<0.001			1.000
Yes	101 (2.4%)	337 (7.3%)		101 (2.4%)	101 (2.4%)	
No/NA	4099 (97.6%)	4254 (92.7%)		4099 (97.6%)	4099 (97.6%)	
**Brain**			<0.001			1.000
Yes	40 (1.0%)	86 (1.9%)		40 (1.0%)	40 (1.0%)	
No/NA	4160 (99.0%)	4505 (98.1%)		4160 (99.0%)	4160 (99.0%)	

PSM: propensity score-matching.
